# Long‐Term Survival Following Nivolumab Treatment in Pulmonary Tumour Thrombotic Microangiopathy Associated With Cancer of Unknown Primary: A Case Report

**DOI:** 10.1002/rcr2.70763

**Published:** 2026-09-30

**Authors:** Kosuke Fujishima, Jun Osugi, Satoshi Noma, Mitsunori Higuchi, Hiroyuki Suzuki

**Affiliations:** ^1^ Department of Thoracic Surgery General South Tohoku Hospital Koriyama Fukushima Japan; ^2^ Department of Thoracic Surgery, Aizu Medical Center Fukushima Medical University Aizuwakamatsu Fukushima Japan; ^3^ Department of Chest Surgery Fukushima Medical University Fukushima Fukushima Japan; ^4^ Department of Respiratory Medicine General South Tohoku Hospital Koriyama Fukushima Japan

**Keywords:** cancer of unknown primary, immune checkpoint inhibitor, pulmonary tumour thrombotic microangiopathy, tree‐in‐bud sign

## Abstract

Pulmonary tumour thrombotic microangiopathy (PTTM) is a rare but frequently fatal condition characterized by rapidly progressive pulmonary hypertension and right heart failure caused by tumour microemboli in the pulmonary vasculature. We report the case of a 64‐year‐old man who presented with rapidly progressive respiratory failure. Chest computed tomography demonstrated centrilobular nodules and a tree‐in‐bud (TIB) pattern. Pulmonary perfusion scintigraphy and transthoracic echocardiography suggested pulmonary hypertension with multiple peripheral perfusion defects, raising strong clinical suspicion for PTTM. Transbronchial lung biopsy (TBLB) revealed TTF‐1–positive adenocarcinoma, although the primary tumour site remained undetermined. Based on these findings, clinically suspected PTTM was diagnosed and nivolumab monotherapy was promptly initiated as systemic treatment for the underlying malignancy. Despite transient severe deterioration, the patient's respiratory function improved markedly, and he has remained progression‐free for more than 3 years. This case highlights the importance of early clinical recognition of PTTM and prompt initiation of appropriate systemic therapy, even before definitive identification of the primary tumour when clinically warranted.

## Graphical abstract

Pulmonary tumour thrombotic microangiopathy (PTTM) is a rapidly fatal condition that is rarely diagnosed during life. Early clinical recognition and prompt nivolumab treatment resulted in long‐term survival in a patient with cancer of unknown primary‐associated PTTM.

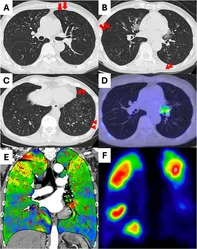

## 1 Introduction

Pulmonary tumour thrombotic microangiopathy (PTTM) is a rare and frequently fatal condition characterized by rapidly progressive respiratory failure and pulmonary hypertension resulting from the occlusion of peripheral pulmonary arteries by tumour microemboli. The clinical course is typically fulminant, and most cases are diagnosed postmortem [1]. Antemortem diagnosis and treatment of PTTM remain exceedingly rare, particularly in cases resulting in long‐term survival. To date, only two cases have been reported in which patients survived for more than 3 years after symptom onset [2, 3]. In this report, we describe a case of clinically diagnosed PTTM with progression‐free survival for more than 3 years following treatment with nivolumab.

## 2 Case Report

A 64‐year‐old man with a 40‐pack‐year smoking history presented with progressive respiratory symptoms. He had received inhaled therapy for bronchial asthma for 2 years. Over the 6 months before presentation, he developed exertional dyspnoea and wheezing. He had neither cough nor haemoptysis, and his symptoms rapidly worsened before consultation. There were no clinical or laboratory findings suggestive of disseminated intravascular coagulation (DIC). Chest CT demonstrated diffuse centrilobular nodules with a TIB pattern accompanied by mild bilateral ground‐glass opacities (GGOs) (Figure 1A–C). Although initially treated for presumed asthma exacerbation with infection, his respiratory status deteriorated. Laboratory testing showed no marked inflammatory response, whereas serum carcinoembryonic antigen (CEA) was elevated (Table 1). Although infectious bronchiolar diseases, including miliary tuberculosis, were considered in the differential diagnosis, microbiological and serological investigations did not support an infectious aetiology. Together with the elevated CEA level and atypical radiological findings, these findings raised suspicion for a noninfectious malignant process. To evaluate possible tumour‐associated vascular involvement, FDG‐PET was performed, revealing mild uptake in a pulmonary ground‐glass lesion without extrathoracic abnormalities (Figure 1D). Within 2 weeks, his respiratory condition rapidly deteriorated, requiring urgent hospitalization for hypoxemia.

**FIGURE 1:**
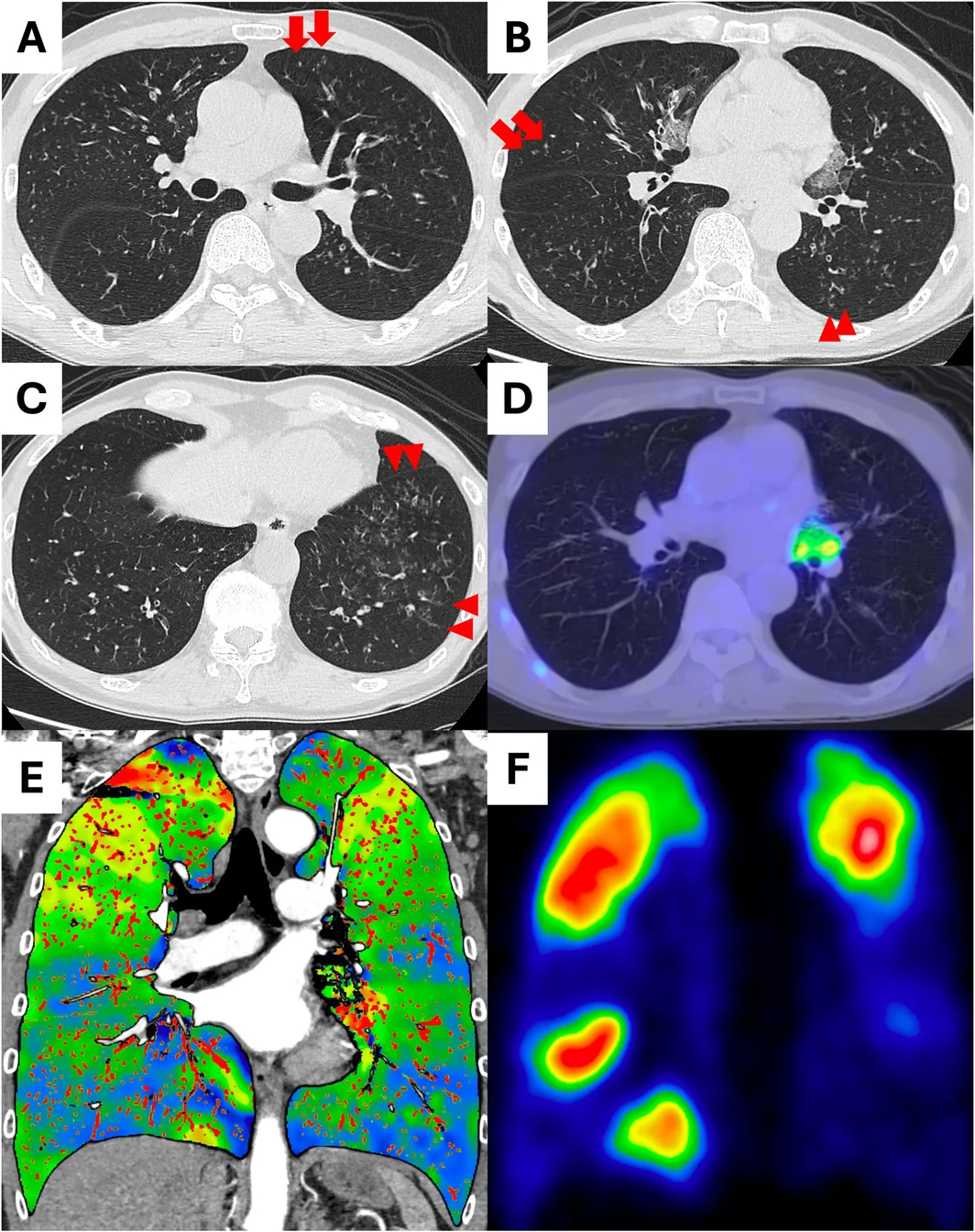
(A–C) Chest CT showing diffuse centrilobular nodules (solid arrows), a tree‐in‐bud pattern (red pointers) and ground‐glass opacities in both lungs. (D) Positron emission tomography using 18F‐fluorodeoxyglucose (FDG‐PET) demonstrating high FDG uptake (SUV 5.4) in the left hilar ground‐glass opacity (GGO). (E) Contrast‐enhanced chest CT revealing no evidence of pulmonary thromboembolism, but decreased perfusion in both lungs. (F) Lung scintigraphy using 99mTc‐MAA demonstrating multiple non‐segmental perfusion defects.

**TABLE 1:** Laboratory examination.

Examination	Result	Normal range
Haematology		
WBC	8910	3900–9700 (/μL)
Neutro.	68.1	38.5–80.5 (%)
Lymp.	19.5	16.5–49.5 (%)
Eos.	7.1	0–8.5 (%)
Bas.	1	0–2.5 (%)
Mon.	4.3	2–10 (%)
RBC	505	438–577 (×10^4^/μL)
Hb	15.9	13.6–18.3 (g/dL)
Hct	47.4	40.4–51.9 (%)
Plt	31.2	14–37.9 (×10^4^/μL)
Coagulation		
PT‐INR	1.07	0.85–1.15
APTT	28.8	24.3–38.9 (s)
D‐dimer	< 0.5	< 1.0 (μg/mL)
Biochemistry		
CRP	0.14	< 0.3 (mg/dL)
KL‐6	245	0–499 (U/mL)
CEA	**34.7**	0–5.0 (ng/mL)
CA19‐9	9	< 37 (U/mL)
CYFRA	1.4	< 2.8 (ng/mL)
CA125	8	< 35 (U/mL)
NSE	12.4	< 16.3 (ng/mL)
SLX	**41**	< 38 (U/mL)
SCC	**5.4**	0–1.5 (ng/mL)
PRoGRP	31.3	< 81.0 (pg/mL)
T‐SPOT.TB	Negative	ー
MAC antibody	< 0.5	< 0.7 (U/mL)
QFT‐GLD	Negative	ー
β‐D‐glucan	8.4	< 20 (pg/mL)
BNP	7.5	< 18.4 (pg/mL)
PCT	0.02	< 0.05 (ng/mL)

*Note:* Bold values indicate values outside the normal reference range.

Abbreviations: APTT, activated partial thromboplastin time; Bas, basophil; BNP, brain natriuretic peptide; CA125, cancer antigen 125; CA19‐9, carbohydrate antigen 19–9; CEA, carcinoembryonic antigen; CRP, C‐reactive protein; CYFRA, cytokeratin 19 fragment; Eos, eosinophil; Hb, haemoglobin; Hct, haematocrit; KL‐6, Krebs von den Lungen‐6; Lymp, lymphocyte; MAC antibody, Mycobacterium avium complex antibody; Mon., monocyte; Neutro, neutrophil; NSE, neuron‐specific enolase; PCT, procalcitonin.; Plt, platelet; ProGRP, pro‐gastrin‐releasing peptide; PT‐INR, prothrombin time – international normalized ratio; QFT‐GLD, QuantiFERON‐TB Gold; RBC, red blood cell; SCC, squamous cell carcinoma antigen; SLX, sialyl Lewis X antigen; T‐SPOT.TB, T‐cell spot test for tuberculosis; WBC, white blood cell; β‐D‐glucan, beta‐D‐glucan.

The post‐admission clinical course is summarized in Figure 2. Contrast‐enhanced chest CT showed no pulmonary artery thrombi, whereas lung perfusion scintigraphy revealed widespread bilateral perfusion defects. Transthoracic echocardiography demonstrated mild pulmonary hypertension with right ventricular strain. On hospital Day 4, bronchoscopy including bronchoalveolar lavage (BAL) and transbronchial lung biopsy (TBLB) from the left upper lobe was performed. Bacterial and mycobacterial cultures of the BAL fluid were negative, further supporting the exclusion of infectious etiologies. Right heart catheterization was subsequently performed on hospital Day 5. Although the hemodynamic findings did not meet the formal diagnostic criteria for pulmonary hypertension, the rapidly progressive clinical course, right ventricular strain on echocardiography, widespread bilateral perfusion defects on lung perfusion scintigraphy, and pathological findings were collectively considered to be most consistent with clinically suspected PTTM. Biopsy specimens revealed adenocarcinoma, but histopathological examination showed preserved vascular architecture without definitive tumour emboli, precluding pathological confirmation of PTTM (Figure 3A,B). Although a pulmonary origin was suspected, the pathological report indicated that the limited biopsy specimen was insufficient to determine the primary site. Because of the patient's rapidly progressive respiratory deterioration, treatment was prioritized before a complete diagnostic work‐up could be completed. Therefore, a clinical diagnosis of PTTM associated with carcinoma of unknown primary (CUP) was made. Following discussion with the patient and his family, nivolumab monotherapy was selected as systemic treatment for the underlying malignancy and initiated on hospital Day 11.

**FIGURE 2:**
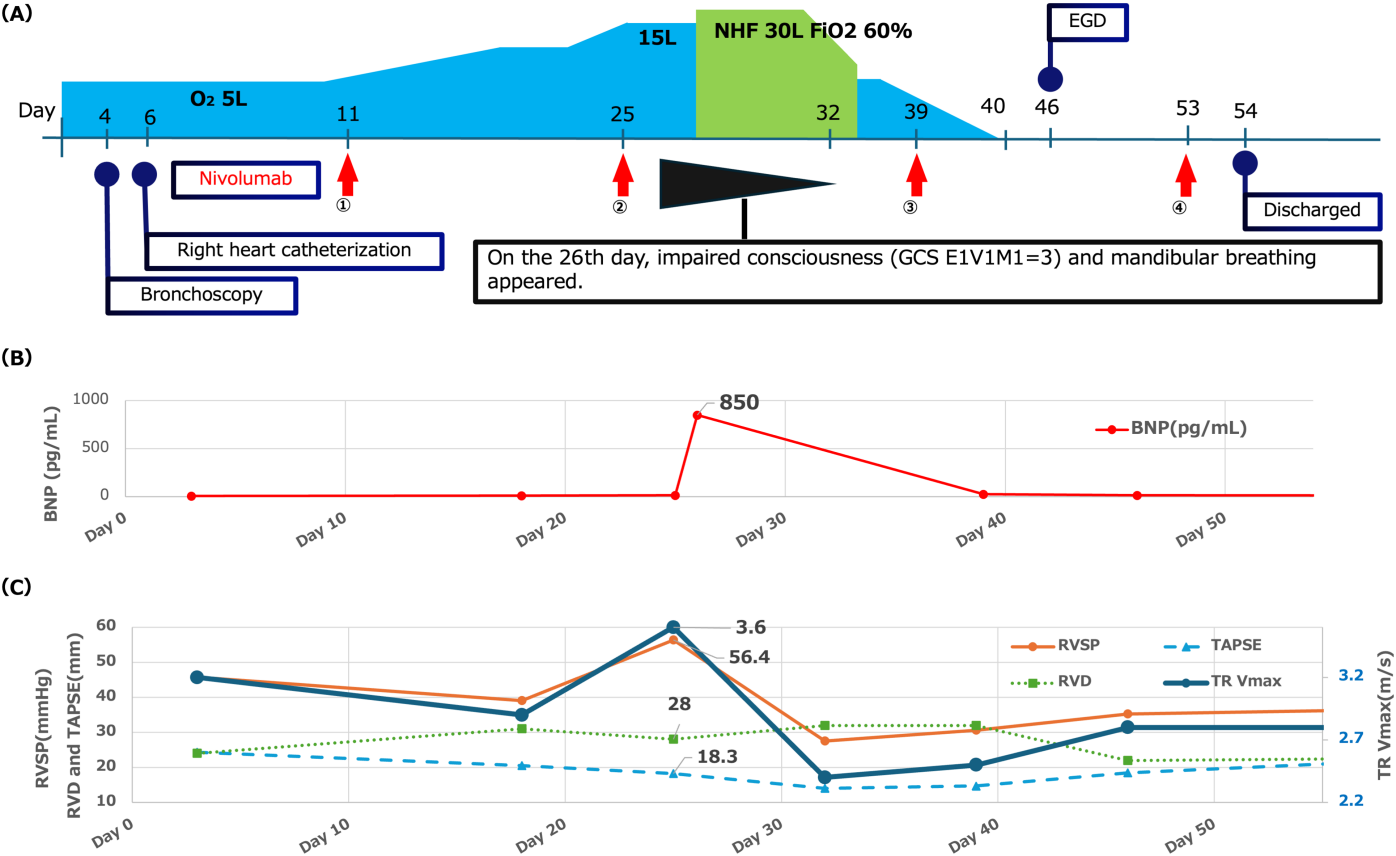
(A) Clinical course during hospitalization. Right heart catheterization: PA 22/10 mmHg (mean PA 16 mmHg), RV 32 mmHg, RA 5 mmHg, PAWP 6 mmHg. Forrester classification: Class I. No tumour cells were detected in pulmonary artery blood. Bronchoscopy: Adenocarcinoma of unknown primary origin was detected in TBLB specimens. Specific mutations: negative; TPS (22C3): 75%. EGD: No tumours were identified. (B) BNP Levels Over Time. Transient elevation and subsequent normalization of serum BNP levels over time. (C) Echocardiographic Parameters Over Time. Dynamic echocardiographic changes reflecting transient right ventricular strain. EGD, esophagogastroduodenoscopy; GCS, Glasgow coma scale; MPA, mean pulmonary arterial pressure; NHF, nasal high flow; PAWP, pulmonary arterial wedge pressure; PH, pulmonary hypertension; RA, right atrium; RV, right ventricle; RVDd, right ventricular end‐diastolic dimension; RVSP, right ventricular systolic pressure; TAPSE, tricuspid annular plane systolic excursion; TBLB, transbronchial lung biopsy; TPS, tumour proportion score; TR Vmax, tricuspid regurgitation peak velocity max.

**FIGURE 3:**
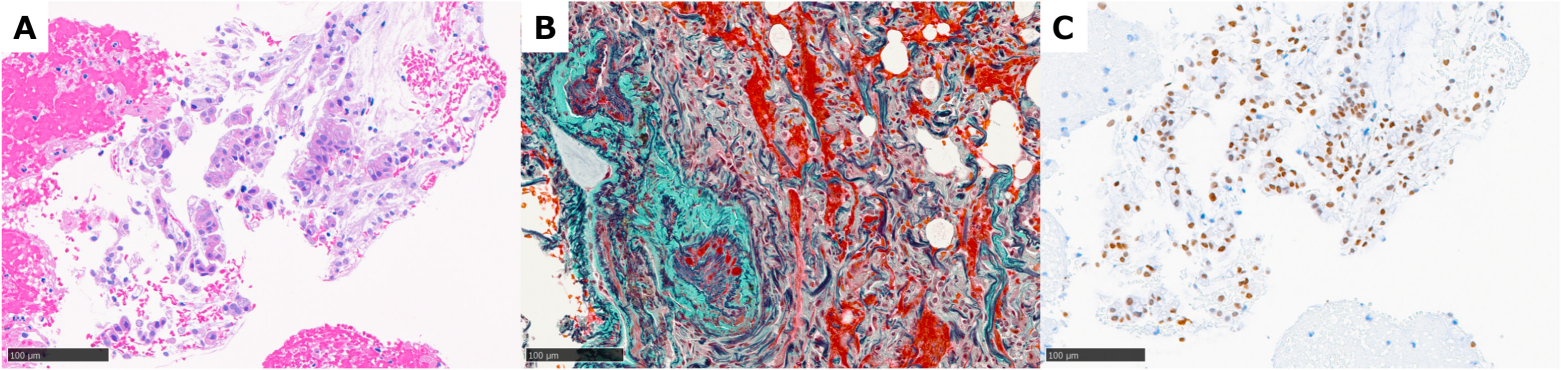
(A) Haematoxylin and eosin staining: A small cluster of atypical glandular epithelial cells with eosinophilic cytoplasm and oval nuclei observed within the alveolar space. (B) Elastica–Masson staining: Preserved vascular architecture without evidence of tumour emboli or fibrin thrombus formation. (C) TTF‐1 immunohistochemistry: Tumour cells showing positive nuclear staining for TTF‐1. Scale bars = 100 μm.

Despite nivolumab initiation, the patient's respiratory condition continued to deteriorate, requiring high‐flow nasal cannula oxygen therapy by Day 14. By Day 26, he developed severe disturbance of consciousness; however, both consciousness and oxygenation improved by Day 29, and he was discharged on Day 54 without neurological or respiratory sequelae. Upper gastrointestinal endoscopy revealed no evidence of malignancy. Immunohistochemical results obtained on Day 16 demonstrated nuclear positivity for thyroid transcription factor‐1 (TTF‐1), although the primary tumour site remained undetermined (Figure 3C). Molecular testing demonstrated no EGFR mutation or ALK or ROS1 rearrangements. PD‐L1 immunostaining (22C3) revealed strong positivity in more than 75% of tumour cells.

During follow‐up, the patient developed several immune‐related adverse events, including thyroid dysfunction, renal dysfunction and hepatic dysfunction. Despite these toxicities, nivolumab was continued with close monitoring. After seven cycles, treatment was discontinued because of Grade 3 pneumonitis. Serial serum CEA measurements showed a progressive decline from 34.7 ng/mL before treatment to within the normal range 2 months after discharge, and the level remained normal throughout follow‐up. The patient remains progression‐free for more than 3 years after disease onset.

## 3 Discussion

PTTM is a rare and often fatal condition characterized by rapidly progressive respiratory failure and pulmonary hypertension. Most cases are diagnosed postmortem, and survival after symptom onset is typically limited to a few weeks [1]. Only two previously reported cases survived longer than 3 years after symptom onset [2, 3]. The present case is exceptional because PTTM was clinically diagnosed, systemic treatment was initiated before definitive identification of the primary tumour, and durable survival for more than 3 years was achieved.

Early diagnosis of PTTM is difficult because its clinical and radiological findings are nonspecific. Ground‐glass opacities and centrilobular nodules are frequently observed on chest CT, whereas pulmonary artery thrombi are usually absent [1]. In the present case, widespread bilateral perfusion defects strongly supported the clinical diagnosis of PTTM despite the absence of pulmonary artery thrombi on contrast‐enhanced CT. Importantly, the diagnosis was based on the integration of the rapidly progressive clinical course, imaging findings, pathological examination and hemodynamic assessment rather than any single diagnostic test. Right heart catheterization was performed early in the clinical course, which may explain why the mean pulmonary artery pressure had not yet reached the diagnostic threshold for pulmonary hypertension. The overall clinical findings were therefore considered most consistent with clinically suspected PTTM. We acknowledge that the absence of pathological confirmation represents a limitation of this clinical diagnosis. Pathological confirmation of PTTM using TBLB is challenging because of sampling error and the limited size of biopsy specimens [1].

The TIB pattern is classically associated with infectious bronchiolar disease, including tuberculosis and nontuberculous mycobacterial infection. However, neoplastic pulmonary emboli can also produce a TIB appearance, referred to as the ‘vascular TIB’ pattern [4]. In the present case, microbiological investigations, including bronchoalveolar lavage cultures, did not support an infectious aetiology. Together with the elevated serum CEA level and rapidly progressive clinical course, these findings suggested a malignant vascular process rather than infection. Serum CEA decreased promptly after initiation of systemic therapy and remained within the normal range during follow‐up, consistent with sustained disease control. The mild bilateral GGOs were also considered to represent tumour‐related rather than infectious pulmonary changes. Although their pathological basis could not be confirmed, previous pathological studies have suggested that such GGOs may reflect alveolar wall thickening caused by tumour‐related interstitial or vascular involvement [5]. Although diffuse alveolar involvement may also have contributed to hypoxemia, pulmonary vascular obstruction was considered the predominant mechanism, based on the widespread bilateral perfusion defects on lung perfusion scintigraphy and the relatively mild extent of the GGOs on CT. Recognition of this vascular‐TIB pattern may facilitate earlier clinical suspicion of PTTM; however, this finding should always be interpreted in conjunction with the clinical presentation and other radiological findings.

Although TTF‐1 positivity and the pulmonary lesion on CT strongly suggested a pulmonary origin, the limited biopsy specimen precluded additional immunohistochemical evaluation, including Napsin A, and therefore did not permit definitive identification of the primary site. Because gastric cancer remains the malignancy most frequently associated with PTTM [1], upper gastrointestinal endoscopy was performed after clinical stabilization; however, no primary lesion was identified. FDG‐PET also demonstrated no clinically relevant extrapulmonary primary tumour. Previous reports have shown that identification of the primary tumour may remain challenging in patients with PTTM despite extensive clinical evaluation [6]. Therefore, although a pulmonary origin was considered most likely, the tumour was classified as CUP because definitive identification of the primary site was not possible. When PTTM is strongly suspected clinically, prompt treatment of the underlying malignancy may be justified even before definitive identification of the primary site.

Currently, no standard treatment has been established for PTTM, and the prognosis remains extremely poor. Reported therapeutic approaches include systemic anticancer therapy, molecular‐targeted therapy, anticoagulation, corticosteroids, pulmonary vasodilators, and, in selected cases, imatinib, although the available evidence is limited to case reports and small case series [1]. Notably, the two previously reported long‐term survivors both achieved marked responses of the underlying malignancy following systemic anticancer therapy, which was accompanied by improvement of PTTM and prolonged survival [2, 3]. Similarly, the present patient achieved durable disease control following systemic therapy. As nivolumab was administered to treat the underlying malignancy rather than PTTM itself, the favourable clinical course was considered to reflect effective tumour control, with subsequent improvement of clinically suspected PTTM. The high PD‐L1 expression observed in this patient may have partly contributed to the favourable response to nivolumab.

These findings suggest that early recognition of PTTM and effective treatment of the underlying malignancy may be key to long‐term survival.

## Author Contributions

**Kosuke Fujishima:** writing – original draft. **Jun Osugi:** writing – review and editing. **Satoshi Noma:** writing – review and editing. **Mitsunori Higuchi:** writing – review and editing. **Hiroyuki Suzuki:** writing – review and editing.

## Funding

The authors have nothing to report.

## Consent

The authors declare that written informed consent was obtained for the publication of this manuscript and accompanying images using the consent form provided by the Journal.

## Conflicts of Interest

The authors declare no conflicts of interest.

## Data Availability

The data that support the findings of this study are available on request from the corresponding author. The data are not publicly available due to privacy or ethical restrictions.
